# Immunometabolic reprogramming in diabetic osteomyelitis: from mechanisms to therapeutics

**DOI:** 10.3389/fcimb.2025.1606317

**Published:** 2025-10-24

**Authors:** Hui Zhang, Zi-Shan Fu, Ying Zhou, Song-Nan Wang, Si-Ying Ye, An-Na Wang, Jun-Tong Liu

**Affiliations:** Liaoning University of Traditional Chinese Medicine, Shenyang, China

**Keywords:** diabetes mellitus, osteomyelitis, immunometabolic reprogramming, mechanisms, therapeutics

## Abstract

Diabetes mellitus (DM) is a globally prevalent metabolic disorder characterized by impaired immune function due to poor glycaemia control, significantly increasing the risk of osteomyelitis. The occurrence of bone infection not only compromises patients’ quality of life but also poses substantial challenges in clinical management. Recent studies have identified immunometabolic reprogramming as a pivotal player in the pathogenesis and progression of diabetic osteomyelitis. This reprogramming not only disrupts immune cell functionality but also modulates the local microenvironment, thereby impairing bone repair processes. Although preliminary research has explored the underlying mechanisms, a comprehensive understanding of the precise role of immunometabolic reprogramming and its potential therapeutic targeting in diabetic osteomyelitis remains elusive. This review synthesizes current advances in immunometabolic reprogramming within diabetic osteomyelitis, elucidates its biological mechanisms, and proposes novel intervention strategies to inform clinical practice and inspire future therapeutic development.

## 1 Introduction

Diabetes mellitus (DM) is a chronic metabolic disorder affecting hundreds of millions of people worldwide. Its prevalence continues to rise, making it a major global public health challenge. Diabetic patients often develop various complications, among which bone infections, such as osteomyelitis, are one of the most common and serious. Diabetic osteomyelitis is a severe complication frequently encountered in diabetic patients. Its pathogenesis is closely associated with immune dysfunction under hyperglycemic conditions. Hyperglycemia not only impairs the function of immune cells but also leads to persistent activation of inflammatory responses, thereby hindering the effective resolution of inflammation and resulting in bone destruction and refractory infection (Frazee, 2024). The occurrence of osteomyelitis in diabetic patients significantly impairs quality of life and may lead to serious outcomes such as amputation. Therefore, a deeper understanding of its underlying mechanisms and corresponding treatment strategies is of great importance.

In recent years, increasing attention has been paid to the role of immunometabolic reprogramming in diabetic osteomyelitis. The metabolic state of immune cells directly influences their function, thereby affecting anti-infective mechanisms and bone healing processes. For example, under chronic hyperglycemia, immune cells in diabetic patients often exhibit metabolic dysfunction, leading to impaired immune responses and increased susceptibility to infection (Favoino et al., 2021; Zhang et al., 2021; Osório et al., 2023). Studies have shown that deep bone ulcers and inflammatory wounds in diabetic patients are significant risk factors for osteomyelitis, highlighting the crucial role of the local immunometabolic microenvironment in the pathogenesis of infection.

Furthermore, research has revealed that diabetic patients exhibit distinct immunometabolic reprogramming features during infection. For instance, macrophages in diabetic patients may adapt to the inflammatory environment by enhancing glycolysis and fatty acid oxidation. This metabolic shift not only alters immune cell behavior but may also promote chronic inflammation and bone tissue damage (Roverato et al., 2021). Thus, investigating the mechanisms of immunometabolic reprogramming in diabetic osteomyelitis may provide new avenues for treatment.

Conventional management of diabetic osteomyelitis primarily includes antibiotic therapy and surgical intervention (Fountas et al., 2024). However, with growing insights into immunometabolic reprogramming, novel therapeutic strategies are emerging. These advances underscore the importance of personalized treatment and multidisciplinary collaboration in clinical practice.

## 2 Diabetic osteomyelitis

**Figure 1:**
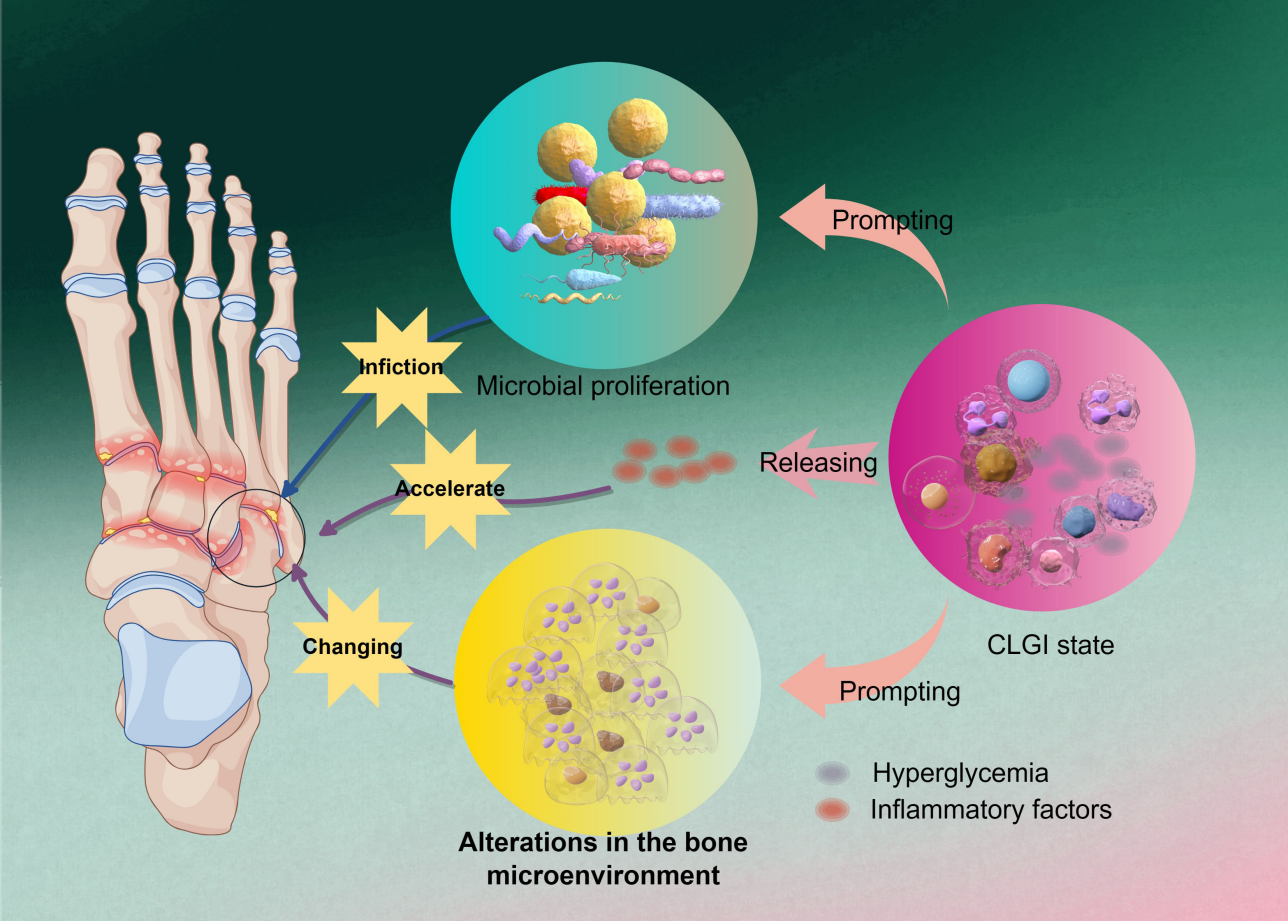
The initiating factor in the pathogenesis of DO. Hyperglycemia exacerbates osteomyelitic progression by inducing a chronic low-grade inflammatory state and releasing inflammatory factors, which alter the bone microenvironment to accelerate microbial proliferation and further amplify pathological alterations in bone tissue, ultimately establishing a self-perpetuating vicious cycle.

Foot infections in diabetic patients often present as chronic ulcers and osteomyelitis, particularly in cases with restricted blood flow. Studies indicate that the incidence of foot osteomyelitis in diabetic patients can be as high as 30% (Zhan et al., 2023). Up to 25% of individuals with diabetes will develop a foot ulcer in their lifetime, and untreated ulcers may progress to bone infection, ultimately leading to amputation (Jaroenarpornwatana et al., 2023). The pathogenesis of diabetic osteomyelitis is complex, involving multiple physiological and pathological factors—including a diabetes-related low-grade inflammatory state, abnormal bone metabolism, and bacterial infection—as illustrated in **Figure 1**.

### 2.1 Chronic low-grade inflammation

A hyperglycemic environment impairs immune cell function, particularly diminishing chemotaxis, oxidative burst, and complement activation in neutrophils, thereby increasing susceptibility to various infections in diabetic patients (Kim and Choi, 2025). Under such conditions, immune cells are unable to effectively recognize and eliminate invading pathogens, leading to persistent bacterial infection in bone tissues. Diabetes is fundamentally a metabolic disorder characterized by the accumulation of excess sugars, lipids, and amino acids in the blood and organs, placing patients in a chronic low-grade inflammatory (CLGI) state. This condition resembles “inflammaging” observed in aging and directly compromises immune cell function (Urszula et al., 2024; Uthaya Kumar et al., 2024). CLGI not only affects glycemic control and induces immune dysfunction, increasing the risk of infection, but also exacerbates diabetes-related complications such as diabetic foot and diabetic nephropathy. Clinically, diabetic foot infection (DFI) is one of the most common complications among diabetic patients, and severe infections including DFI-related osteomyelitis are major causes of hospitalization and limb amputation (Frazee, 2024).

Immune cells in diabetic patients exhibit significant impairments in proliferation and cytokine secretion, particularly in macrophage and T-cell function. Under CLGI conditions, T cells and B cells display features of cellular senescence (Wei et al., 2021; Saadh et al., 2025). Macrophages show reduced ability to recognize and clear pathogens, manifested by weakened phagocytosis and dysregulated cytokine secretion (Pan et al., 2024; Saadh et al., 2025). Consequently, diabetic patients are notably more susceptible to infections, especially those caused by certain bacteria and viruses (Alexander et al., 2024; Uthaya Kumar et al., 2024). Thus, CLGI can be regarded as both the pathological basis and initiating factor in the development of diabetic osteomyelitis.

### 2.2 Alterations in the bone tissue microenvironment

In hyperglycemic mice infected with *Staphylococcus aureus*, the bacterial load within the bone is significantly increased, and the extent of bone destruction is more severe compared to normoglycemic mice, indicating exacerbated osteomyelitis symptoms (Butrico et al., 2023). This suggests that hyperglycemia not only promotes bacterial proliferation but also aggravates infection-induced bone damage. Within the context of chronic low-grade inflammation (CLGI), an imbalance in inflammatory mediators and cytokines disrupts the dynamic equilibrium between bone resorption and bone formation. For instance, infiltrating inflammatory cells such as macrophages secrete large quantities of pro-inflammatory factors within the bone marrow. These factors promote osteoclastogenesis, enhance bone resorption, and inhibit osteoblast function, thereby exacerbating osteoporosis and increasing the risk of fracture (Heng et al., 2023; Chen J. et al., 2025). Furthermore, such inflammatory cytokines may also induce osteoblast apoptosis, impairing the bone’s regenerative capacity (Zhang F. et al., 2024; Hu et al., 2025). For example, macrophages in the bone marrow of diabetic patients often exhibit a pro-inflammatory phenotype, secreting substantial amounts of tumor necrosis factor-alpha (TNF-α) and interleukin-6 (IL-6). The release of these cytokines promotes osteoclast activation, accelerating bone resorption and impairing bone repair and regeneration (Yu et al., 2020; Hu et al., 2025).

Thus, within the setting of diabetic osteomyelitis, alterations in the bone microenvironment constitute a complex and multifactorial process. Firstly, imbalanced bone metabolism—a common pathological feature in diabetes—is characterized by enhanced bone resorption and impaired bone formation (Bakhtiyari et al., 2023; Perera Molligoda Arachchige and Verma, 2024). This ultimately leads to aggravated bone destruction and reduced bone density, compromising bone structural integrity (Heng et al., 2023). Additionally, diabetic patients often exhibit microangiopathy in bone tissue, resulting in local ischemia that exacerbates tissue damage and increases infection risk. Impaired blood supply due to microvascular complications places bone tissue at a disadvantage during repair and regeneration, making infections more likely to occur and worsen (Perera Molligoda Arachchige and Verma, 2024). Therefore, from a pathological perspective, changes in the bone microenvironment can be viewed as an extension of CLGI, representing its structural impact on bone. The destruction of bone structure allows pathogenic microorganisms to directly enter the bone marrow through damaged sites, triggering the onset of diabetic osteomyelitis.

### 2.3 Characteristics of pathogenic microorganism infection

Common pathogenic microorganisms responsible for osteomyelitis include *Staphylococcus aureus*, *Pseudomonas aeruginosa*, and *Escherichia coli*. Among these, *Staphylococcus aureus* is considered the most prevalent and virulent pathogen. The persistence of its infection is closely linked to its metabolic characteristics. *S. aureus* facilitates its survival and proliferation through the production of various toxins and enzymes. For instance, it can secrete β-lactamase, which confers resistance to penicillin-based antibiotics, thereby contributing to persistent infection (Urish and Cassat, 2020). Polymicrobial infections are highly common in osteomyelitis, especially in patients with prolonged antibiotic use or compromised immune function. For example, the proportion of Gram-negative bacteria such as *Pseudomonas aeruginosa* and *Escherichia coli* in mixed infections is gradually increasing, and the drug resistance of these bacteria is continuously strengthening, posing significant challenges to conventional treatment strategies (Ruiz Holgado et al., 2023; Zhang et al., 2023).

Moreover, the formation of microbial biofilms is a critical factor contributing to the persistence of osteomyelitis infections. Biofilms are structures formed by bacterial self-produced polymers on surfaces, which confer strong drug resistance and immune evasion capabilities. Bacteria within biofilms can adapt their metabolic patterns to evade host immune responses and antibiotic treatments, thereby enhancing their survival (Yu et al., 2025). For instance, *S. aureus* in a biofilm environment can modulate its metabolic pathways to establish a persistent infectious niche within bone and surrounding tissues, protecting itself from host immune attacks while increasing tolerance to antibiotics, leading to reduced efficacy of conventional therapies. Furthermore, biofilm formation not only influences pathogen resistance but also significantly exacerbates bone destruction. Bacteria within biofilms secrete various enzymes and toxins that directly contribute to bone degradation and intensify inflammatory responses, thereby worsening the condition of osteomyelitis (Unsworth et al., 2024; Al Ghaithi et al., 2025).

## 3 Immunometabolic reprogramming

The immune system comprises various cell populations, including lymphocytes (T cells and B cells), monocytes, macrophages, and neutrophils, each playing distinct roles in host defense. T cells mediate cellular immunity, while B cells contribute to humoral immunity through antibody production. Macrophages and monocytes act as professional phagocytic cells, and neutrophils form the primary defense against bacterial infections (Alexander et al., 2024). The metabolic state of immune cells is closely linked to their functions, as summarized in **Table 1** (Pearce and Pearce, 2013), which lists the metabolic profiles of major immune cells and their corresponding purposes and functions. In a resting state, immune cells primarily rely on oxidative phosphorylation for energy production. However, upon activation, they rapidly shift to glycolysis to meet the energy and biosynthetic demands required for proliferation and effector functions (Hu et al., 2022). This metabolic reprogramming is a critical mechanism by which immune cells adapt to environmental changes and regulate their biological functions.

**Table 1:** Main metabolic pathways of immune cells and changes in function after reprogramming.

Immune Cells	Main metabolic pathways	Purpose and function
Resting T Cells/Naive T Cells	Oxidative Phosphorylation	Highly efficient, utilizing minimal nutrients to maintain basic survival and surveillance functions
Activated Effector T Cells	Aerobic Glycolysis	Rapidly produces ATP and biosynthetic precursors to support rapid proliferation and cytokine production (e.g., IFN-γ, TNF-α).
Memory T Cells	Fatty Acid Oxidation	Highly efficient, relies on mitochondrial metabolism to provide sustained energy for long-term survival and rapid reactivation capacity.
M1 Macrophages	Aerobic Glycolysis	Pro-inflammatory state, rapidly produces energy and intermediates to support antibacterial activity and nitric oxide (NO) production.
M2 Macrophages	Oxidative Phosphorylation	Anti-inflammatory/repair state, utilizes fatty acid and glucose oxidation to support tissue repair and arginine metabolism.
Regulatory T Cells	Fatty Acid Oxidation	Supports their suppressive function and self-stability.

In infection and tumor microenvironments, activated T cells upregulate glycolysis to rapidly acquire ATP and precursor molecules necessary for biosynthesis, thereby enhancing their effector functions (Bittman, 2022). This metabolic reprogramming not only affects the energy metabolism of immune cells but also directly regulates their cytokine secretion. For instance, the glycolytic byproduct lactate has been found to modulate immune cell functions, influencing T cell differentiation and activity (Fortuny and Sebastián, 2021). In the tumor microenvironment, the acidic and nutrient-deprived conditions resulting from tumor cell metabolism suppress oxidative phosphorylation in immune cells, further promoting glycolysis and leading to an immunosuppressive state (Talty and Olino, 2021). In addition to glucose metabolism dysregulation, patients with diabetes often exhibit alterations in lipid metabolism (Talty and Olino, 2021) and amino acid metabolism pathways (Mark and Tansey, 2025). For example, M1 macrophages in inflammatory environments enhance fatty acid synthesis and oxidation, which promotes the secretion of pro-inflammatory cytokines (Shanley et al., 2022). In adipose tissue, interleukin-1β (IL-1β) and interleukin-18 (IL-18) are abnormally activated and excessively released (Alghamdi et al., 2023; Yan, 2024), thereby exacerbating inflammatory responses.

In fact, beyond directly mediating inflammatory responses, metabolic dysregulation is itself a key trigger of immune cell metabolic reprogramming, with the two processes often being mutually causal. Inflammatory cytokines, such as tumor necrosis factor-α (TNF-α) and interleukin-6 (IL-6), can regulate the metabolic state of immune cells by activating specific signaling pathways (Khandelwal et al., 2022). Conversely, inflammation-induced insulin resistance impairs cellular glucose utilization and exacerbates metabolic dysregulation. Under inflammatory conditions, cells typically upregulate glycolytic flux to meet increased energy demands—a phenomenon known as the “Warburg effect.” For instance, activated macrophages significantly enhance glucose uptake and glycolytic activity during inflammatory responses to support rapid proliferation and effector functions (Balic et al., 2020). However, this metabolic reprogramming not only disrupts energy homeostasis but also leads to the accumulation of potentially harmful metabolites, such as lactate, forming a pathological positive feedback loop that perpetuates cellular dysfunction (Deng et al., 2025).

## 4 Immunometabolic reprogramming in diabetic osteomyelitis

### 4.1 Cytokines involved in the formation of diabetic osteomyelitis

As previously described, the formation of diabetic osteomyelitis is a complex process involving vascular endothelial dysfunction, abnormal bone metabolism, and bacterial infections. However, its core mechanism can be summarized as the excessive activation of the immune system under a chronic low-grade inflammatory state. In this chronic low-grade inflammatory state, the cytokines that induce diabetic osteomyelitis can be categorized as follows:

#### 4.1.1 Pro-inflammatory cytokines

**Figure 2:**
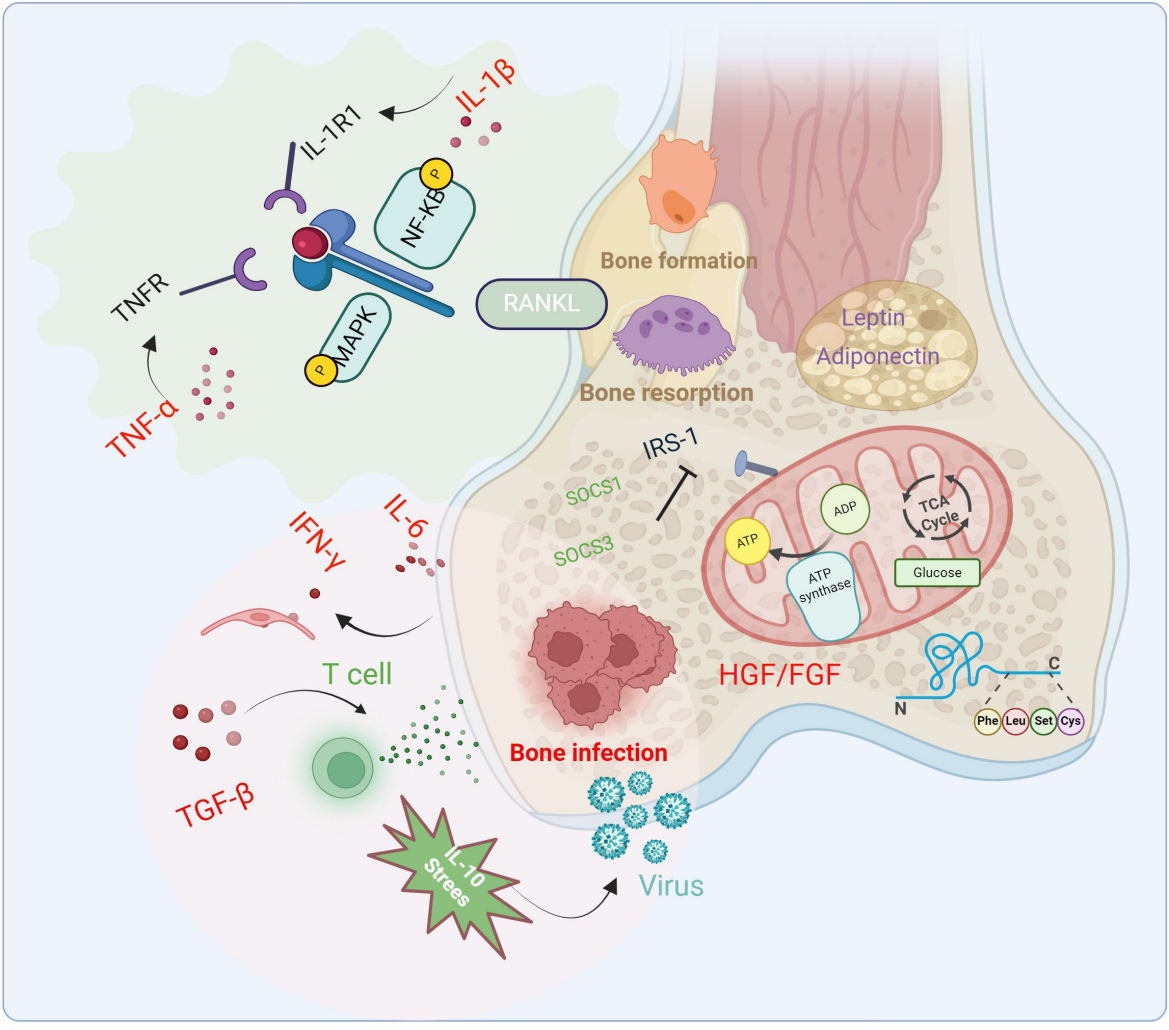
Changes of cytokines, signaling pathways and related immune factors in the pathophysiology of diabetic osteomyelitis. Cytokines and inflammatory factors inhibit bone formation, promote bone resorption and bone infection through glycolysis and fat metabolism, and aggravate the occurrence of diabetic osteomyelitis.

Pro-inflammatory cytokines are the most critical cytokines in inducing diabetic osteomyelitis, with their upregulation being a hallmark of its pathological process. In a persistent inflammatory state, bone cell energy metabolism shifts from oxidative phosphorylation to glycolysis to meet cellular energy demands. This metabolic reprogramming alters the energy supply pathways of bone cells, subsequently affecting their downstream functions, such as increased bone resorption and decreased bone formation. Consequently, metabolic reprogramming in bone cells increases the risk of osteoporosis and bone infections. For instance, IL-1β and TNF-α influence bone cell energy metabolism by regulating glucose uptake and utilization. TNF-α directly activates NF-κB and mitogen-activated protein kinase (MAPK) pathways (e.g., p38, JNK) through TNFR, driving osteoclast differentiation even under low RANKL conditions. IL-1β activates NF-κB and MAPK via IL-1R1, synergizing with TNF-α to promote osteoclastogenesis. IL-1β also mediates TNF-α-induced osteoclast formation (Baud’Huin et al., 2010; O’Brien et al., 2016). IL-6 induces IFN-γ-dependent endothelial cell damage and subsequent IgG loss, ultimately exacerbating bone infections following bone marrow transplantation. Furthermore, IL-6 may aggravate the pathological progression of bone infections by affecting bone metabolism (Li et al., 2022; Luo et al., 2024). In summary, these cytokines can alter the activity and differentiation state of bone cells by influencing glucose and lipid metabolism (**Figure 2**).

#### 4.1.2 Anti-inflammatory cytokines

Compared to pro-inflammatory cytokines, anti-inflammatory cytokines play a more significant important role in diabetic osteomyelitis. In a hyperglycemic state, the accumulation of advanced glycation end products (AGEs) promotes the synthesis of pro-inflammatory cytokines while inhibiting the production of anti-inflammatory cytokines such as IL-10, thereby exacerbating the inflammatory response (Zhao et al., 2024). In a chronic low-grade inflammatory (CLGI) state, the expression of pro-inflammatory cytokines gradually increases, while that of anti-inflammatory cytokines, such as IL-10 and TIPE2, decreases. These anti-inflammatory cytokines are critical for alleviating the progression of diabetic osteomyelitis and accelerating disease recovery. For example, significant downregulation of TIPE2 is closely associated with the progression of diabetic retinopathy (Suo et al., 2021).

As a key anti-inflammatory cytokine in diabetic osteomyelitis, IL-10 inhibits the activity of T cells and macrophages, reducing the production of pro-inflammatory cytokines and thereby mitigating tissue inflammation. In diabetic patients, IL-10 levels are typically low, which may lead to more severe inflammatory and infectious responses, thus impairing the healing process of osteomyelitis (Bui et al., 2022). TGF-β is another critical anti-inflammatory cytokine, widely involved in regulating cell proliferation, differentiation, and immune responses. Studies show that TGF-β promotes fibroblast activation and proliferation, facilitating wound healing, and reduces inflammatory cell infiltration to restore the function of damaged tissues. In diabetic patients, TGF-β regulates the local microenvironment, reducing inflammatory cell infiltration and destructive responses in osteomyelitis (Dormer and Gkotsoulias, 2022). Anti-inflammatory cytokines, such as IL-10 and transforming growth factor-β (TGF-β), can directly induce macrophage polarization toward the M2 phenotype, thereby altering the immune microenvironment. In chronic inflammatory conditions like diabetic osteomyelitis, the generation and function of M2 macrophages are considered important factors in improving disease outcomes. Notably, the functions of anti-inflammatory cytokines extend beyond suppressing inflammation to include participation in tissue remodeling and healing processes (Xie et al., 2020). Insufficient anti-inflammatory cytokines lead to persistent chronic inflammation, impairing wound healing and increasing the risk of osteomyelitis.

#### 4.1.3 Pro-resolving mediators

Specialized pro-resolving mediators (SPMs) are important bioactive lipids, including resolvins, maresins, and lipoxins, primarily derived from the metabolism of polyunsaturated fatty acids (PUFAs), particularly ω-3 and ω-6 fatty acids. Unlike anti-inflammatory cytokines, which directly suppress inflammatory cytokines to regulate the immune microenvironment, SPMs promote inflammation resolution in a more systematic and multi-level manner. In addition to regulating inflammatory responses, SPMs also facilitate tissue repair and suppress excessive immune responses (Rasquel-Oliveira et al., 2023; Liu et al., 2024). Resolvins are divided into E-series and D-series, with E-series resolvins primarily derived from eicosapentaenoic acid (EPA) and D-series resolvins from docosahexaenoic acid (DHA). The synthesis of resolvins requires regulation by specific enzymes, with lipoxygenase and cyclooxygenase being key enzymes in their production (Cebrián-Prats et al., 2022). Additionally, maresins, another important SPM derived from DHA, play a significant role in regulating inflammation and promoting tissue repair. Studies have shown that maresins not only suppress inflammatory responses but also enhance macrophage functions, improving their pathogen clearance capabilities (Rasquel-Oliveira et al., 2023). Furthermore, SPMs reduce the production of pro-inflammatory cytokines and inhibit the activity of pro-inflammatory signaling pathways such as NF-κB, which is crucial for maintaining immune homeostasis and reducing chronic inflammation (Peh and Chen, 2025).

In the context of diabetic osteomyelitis, the lack of effective inflammation resolution mechanisms leads to persistent chronic inflammation, exacerbating disease progression. Thus, promoting inflammation resolution is of significant importance in diabetic osteomyelitis (Zhao et al., 2023; Leuti et al., 2024). For example, SPMs significantly enhance the clearance rate of inflammatory cells and improve tissue healing in diabetic mouse models (Albuquerque-Souza and Dalli, 2025). Diabetic osteomyelitis often involves tissue damage, and SPMs enhance tissue regeneration by promoting cell proliferation and migration, thereby accelerating wound healing and tissue repair (Serhan et al., 2024).

One of the mechanisms by which SPMs ameliorate diabetic osteomyelitis is through modulation of the immune landscape. Resolvins and protectins, as key regulators of macrophage polarization, effectively promote the shift to the M2 phenotype. These M2 macrophages not only secrete anti-inflammatory cytokines but also contribute to repair processes by clearing cellular debris and promoting tissue regeneration (Videla et al., 2023). SPMs also enhance macrophage efferocytosis (the clearance of apoptotic cells), facilitating inflammation resolution and tissue regeneration (Vetter and Saas, 2024). By inducing neutrophil apoptosis, SPMs effectively reduce the release of inflammatory mediators, thereby alleviating inflammation. This process not only aids in tissue repair and reconstruction but also restores normal immune function (Baker and Cantley, 2025). Pro-resolving mediators promote the transition of neutrophils to an anti-inflammatory phenotype, enhancing their clearance functions and suppressing inflammatory responses by altering their metabolic pathways (Psarras and Clarke, 2023). Additionally, certain SPMs reduce tissue damage by inhibiting neutrophil activation, thereby decreasing the production of reactive oxygen species (ROS) and inflammatory cytokines (Albiero and Baragetti, 2025). This multi-level regulation of neutrophils effectively ameliorates the inflammatory state in diabetic osteomyelitis, accelerating the recovery of damaged tissues.

However, the synthesis of SPMs in diabetic patients is suppressed by hyperglycemia (Brennan et al., 2021). Impaired pro-resolving signaling pathways are also a significant cause of defective inflammation resolution in diabetic environments. For instance, macrophages lacking PRMT2 exhibit enhanced inflammatory responses, further exacerbating atherosclerosis development (Vurusaner et al., 2022). Similarly, SIRT6 deficiency has been found to impair macrophage efferocytosis, thereby aggravating persistent inflammation (Ontoria-Oviedo et al., 2022). These findings indicate that the combined effects of impaired SPM synthesis and defective signaling pathways in diabetic environments hinder effective inflammation resolution, ultimately affecting bone tissue health and healing capacity.

### 4.2 Immunometabolic reprogramming of immune cells in diabetic osteomyelitis

**Figure 3:**
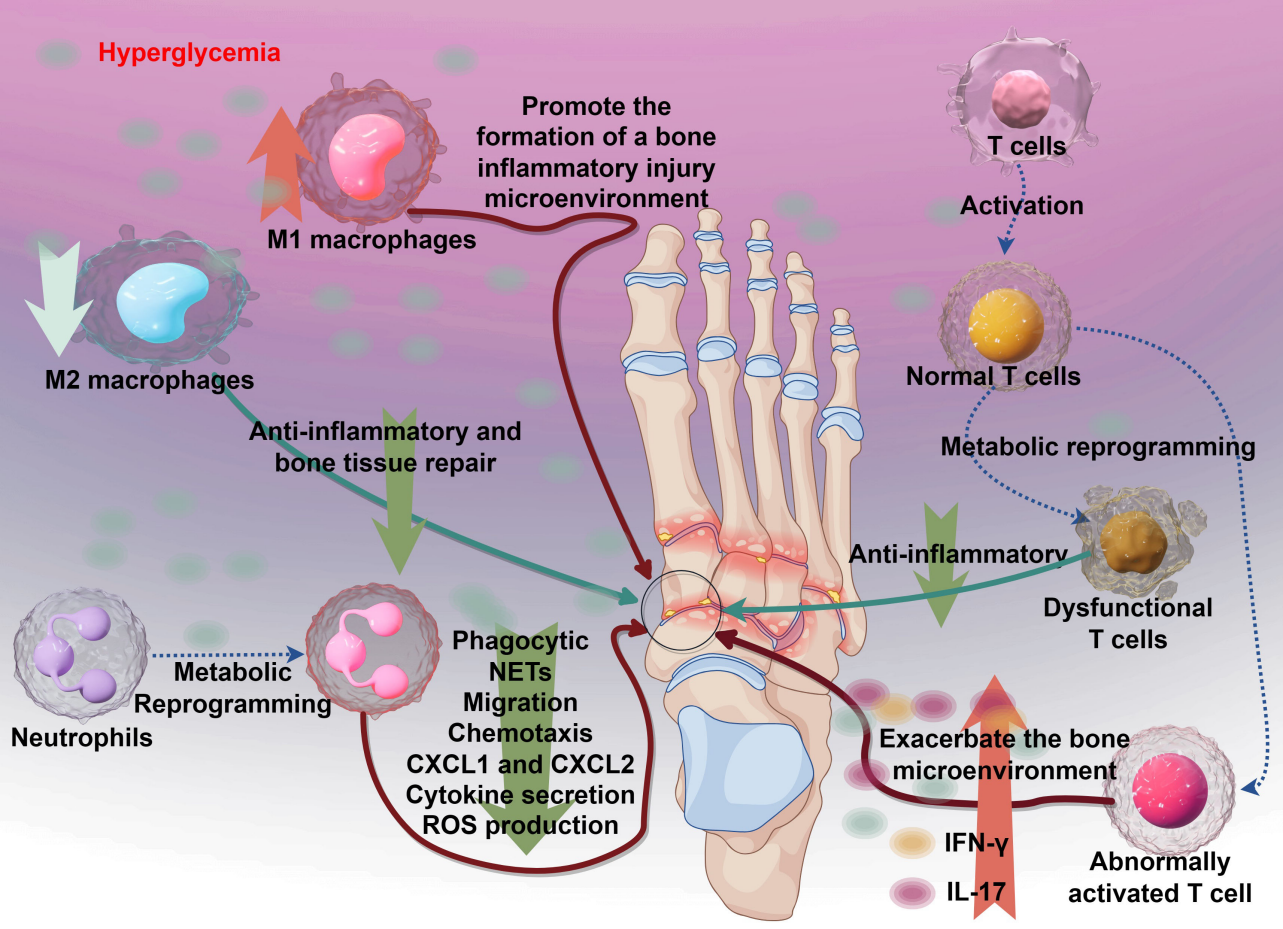
The immune mechanisms underlying diabetic osteomyelitis and the dysregulation of key immune cells. Hyperglycemia initiates the process by promoting the formation of a bone inflammatory injury microenvironment. This leads to aberrant T cell activation, resulting in dysfunctional T cells that exacerbate the bone microenvironment instead of resolving inflammation. Concurrently, metabolic reprogramming alters immune responses: M1 macrophages drive pro-inflammatory reactions, while M2 macrophages contribute to anti-inflammatory and bone tissue repair. Neutrophils exhibit impaired function, with reduced phagocytic activity, aberrant NETS formation, and disrupted migration and chemotaxis (mediated by CXCL1 and CXCL2), alongside enhanced cytokine secretion and ROS production. Collectively, these immune perturbations amplify tissue damage and hinder resolution of infection in diabetic osteomyelitis.

As mentioned above, diabetic osteomyelitis is often a more severe form of diabetic bone infection, accompanied by immune cell metabolic reprogramming, immune system dysfunction, and impaired pathogen clearance, leading to abnormal proliferation of external bacteria at the infection site (Bermejo Olano et al., 2024). Therefore, aside from the microbial infection factors inherent to osteomyelitis, the core factor contributing to the disease is immune system dysfunction, which involves changes in the phenotypes of multiple immune cells (see **Figure 3**).

#### 4.2.1 M1/M2 polarization of macrophages

Macrophages in the immune system exhibit high plasticity, differentiating into pro-inflammatory M1 or anti-inflammatory M2 macrophages under the mediation of various cytokines in the immune microenvironment (Davis et al., 2013). Glycolysis is critical for M1 activation and serves as the core pathway for the antibacterial and antitumor functions of M1 macrophages (Corrado et al., 2020). Under hyperglycemic conditions, bone marrow-derived macrophages and monocytes exhibit enhanced glycolysis and oxidative responses, with altered oxidative phosphorylation (OXPHOS) and modified hexokinase II (HK2) activity in M1 macrophages. Although M1 polarization contributes to microbial killing, excessive M1 activation exacerbates bone destruction in diabetic osteomyelitis. Additionally, bacterial infections in diabetic osteomyelitis may further stimulate these pathways by activating nuclear factor κB (NF-κB) in B cells. Persistent inflammation at the infection site recruits more macrophages and promotes M1 polarization (Case et al., 2021; Liu H. et al., 2023), forming a vicious cycle. Given that the primary feature of M1 polarization is the upregulation of glycolytic enzymes, including phosphofructokinase (PFK1 and PFK2) isoforms, PFK-catalyzed reactions are irreversible key steps in glycolysis. Inhibiting PFK2 can reduce the expression of inducible nitric oxide synthase (iNOS) and cyclooxygenase-2 (COX2), ultimately suppressing M1 macrophage polarization. Phosphofructokinase-2/fructose-2,6-bisphosphatase 3 (PFKFB3) is also an important regulatory target, and its inhibition significantly suppresses downstream glycolytic reactions and M1 macrophage activation (Cui et al., 2015; De Rosa et al., 2015).

In contrast, M2 macrophages preferentially utilize oxidative phosphorylation to maintain their anti-inflammatory and reparative functions (Li et al., 2021). Studies indicate that M2 polarization enhances collagen deposition, angiogenesis, and wound healing. For example, M2 macrophage-derived exosomes (M2D-Exos) promote osteogenic differentiation both *in vitro* and *in vivo*. M2D-Exos containing miR-5106 may inhibit salt-inducible kinase 2 (SIK2) expression via the cAMP response element-binding protein (CREB) signaling pathway to stimulate angiogenesis (Chen et al., 2023). It has also been reported that M2D-Exos reduce adipogenic differentiation of bone marrow stromal cells (BMSCs) via the miR-690/IRS-1/TAZ axis (Li et al., 2021), suggesting that M2D-Exos promote bone repair through metabolic reprogramming pathways (MacKenna et al., 2022). However, in diabetic osteomyelitis, M2 macrophages are not predominant, leading to a significant reduction in their ability to clear apoptotic cells and suppress inflammatory damage induced by IL-4, IL-10, IL-13, and TGF-β. This manifests in the body as T lymphocyte recruitment and granuloma formation in bone marrow tissue (Lawrence and Gilroy, 2007; Murray et al., 2014), further activating downstream chronic innate immune activation and low-grade inflammation (Hotamisligil, 2006; Alghamdi et al., 2023), accelerating the progression of diabetic osteomyelitis.

#### 4.2.2 Neutrophil chemotaxis

Neutrophils are the most abundant white blood cells in the immune system and possess multiple critical immune functions. During infection and inflammation, neutrophils participate in host defense through mechanisms such as chemotaxis, phagocytosis (Liu J. et al., 2023), oxidative bursts (Guo et al., 2024), and neutrophil extracellular trap (NET) formation (Abuaita et al., 2021). The multifaceted roles of neutrophils in infection defense and their importance in maintaining immune homeostasis make them key targets for studying immune-related diseases. Studies indicate that neutrophil dysfunction may contribute to the development of various diseases, such as autoimmune disorders and chronic inflammation (Simmons et al., 2025). Chemotaxis is a critical mechanism by which neutrophils respond to infections, as they are attracted to infection sites by chemical signals (e.g., bacterial compounds or inflammatory mediators), enabling rapid aggregation and function (Trivedi et al., 2021). During this process, neutrophil surface receptors recognize pathogens and their products, initiating intracellular signaling pathways that enhance antibacterial capabilities.

As the first line of defense in the immune system, neutrophils rely heavily on metabolic pathways during immune responses. Regarding glycolysis, studies show that activated neutrophils primarily depend on glycolysis to generate energy to meet the demands of rapid responses. Activated neutrophils increase glucose uptake and metabolism, promoting energy production and reactive oxygen species (ROS) generation, thereby enhancing their bactericidal capacity (Toller-Kawahisa and O’Neill, 2022). Additionally, glycolysis not only provides energy but also enhances antibacterial effects by promoting NET formation (Calo et al., 2025). In terms of oxidative phosphorylation, although neutrophils are traditionally considered reliant on glycolysis, recent studies have found that they can also utilize oxidative phosphorylation under specific conditions. This shift plays a critical role in neutrophil differentiation and functional regulation. For example, mature neutrophils switch to oxidative phosphorylation-dominated metabolism under hypoxic conditions to adapt to the tumor microenvironment (Huang et al., 2025). This metabolic adaptability affects not only neutrophil energy metabolism but also their role in tumor progression. Fatty acid oxidation is another critical component of neutrophil metabolism, particularly in chronic inflammation and tumor microenvironments. Neutrophils can rely on fatty acid oxidation to support energy supply, especially in nutrient-limited conditions (Calo et al., 2025). Moreover, fatty acid oxidation is closely associated with neutrophil antibacterial activity, chemotaxis, and NET formation, and these functions may be altered in different pathological states.

Neutrophil function is largely regulated by their metabolic state. Metabolic reprogramming enables these cells to adjust energy production and functional performance in response to microenvironmental changes, addressing various physiological and pathological challenges. For instance, during acute infections, neutrophils rapidly generate ATP and ROS through glycolysis to enhance pathogen clearance (Jiang et al., 2022). These metabolic changes affect not only energy supply but also cellular signaling and function execution. In chronic inflammation or tumor microenvironments, neutrophil metabolism undergoes significant changes. For example, tumor-associated neutrophils (TANs) often exhibit metabolic reprogramming, relying primarily on enhanced glycolysis and fatty acid oxidation, leading to immunosuppressive functions (Lee et al., 2024). This reprogramming enables neutrophils to protect tumor cells and suppress anti-tumor immune responses, promoting tumor growth and metastasis.

Additionally, metabolic state is closely linked to neutrophil survival and death. Different metabolic pathways regulate neutrophil lifespan through mechanisms such as apoptosis and autophagy. For example, studies suggest that enhanced glycolysis can prolong neutrophil lifespan by delaying apoptosis, a regulation particularly significant in infection and inflammation contexts (Leblanc et al., 2024). In hyperglycemic conditions, neutrophil metabolism undergoes significant changes, primarily characterized by enhanced glycolysis and increased oxidative stress. Elevated intracellular glucose concentrations in hyperglycemia stimulate neutrophils to enhance energy production via glycolysis, a process closely linked to NADPH oxidase activation (Joshi et al., 2020). Studies indicate that hyperglycemia-induced glucose metabolism alterations lead to excessive ROS production by neutrophils, triggering oxidative stress responses that impair neutrophil survival and function (Cázares-Preciado et al., 2024).

Metabolic abnormalities extend beyond enhanced glycolysis to include changes in fatty acid metabolism. In hyperglycemia, neutrophil energy metabolism shifts from primarily glycolysis to fatty acid oxidation, which sustains cellular activity to some extent but may also lead to functional dysregulation. For example, in diabetic patients, metabolic reprogramming significantly weakens neutrophil antibacterial capacity, manifesting as reduced phagocytosis and NET formation (Li et al., 2023; Jolibois et al., 2024). This metabolic reprogramming increases the persistence and severity of inflammatory responses in diabetic patients, making them more susceptible to infections (Sun et al., 2025).

Metabolic abnormalities may also lead to neutrophil dysfunction, characterized by reduced migration, chemotaxis, and cytokine secretion, accelerating the progression of diabetic osteomyelitis. For instance, in hyperglycemia, the expression of chemokines CXCL1 and CXCL2 is suppressed, impairing neutrophil recruitment and the effectiveness of inflammatory responses (Thimmappa et al., 2023). Additionally, reduced ROS production capacity in diabetic neutrophils further weakens their bacterial clearance ability, a phenomenon termed “diabetes-associated immunosuppression” (Darwitz et al., 2024).

#### 4.2.3 T lymphocyte activation

In the resting state, T cells primarily rely on oxidative phosphorylation (OXPHOS) for energy production, a process occurring in mitochondria to support basic physiological functions and maintain metabolic homeostasis. However, upon activation, T cells undergo significant metabolic reprogramming. Studies show that activated T cells enhance glycolysis to meet the energy demands of rapid proliferation and effector functions, favoring glycolysis even under aerobic conditions, a phenomenon known as the Warburg effect (Romero-Carramiñana et al., 2024). This metabolic shift not only supports cell activation but also provides the energy and biosynthetic precursors necessary for cytokine synthesis and proliferation. Through glycolysis, T cells rapidly produce ATP and lactate, with lactate accumulation further influencing the microenvironment and immune responses (Liu et al., 2022). Additionally, the balance between oxidative phosphorylation and glycolysis is critical for T cell function, and its disruption may lead to T cell exhaustion and dysfunction, particularly pronounced in metabolic diseases like diabetes (Cao et al., 2024).

In the diabetic state, hyperglycemia and metabolic dysregulation cause T cell metabolic abnormalities, significantly impairing their activation and effector functions. Studies indicate that T cells in diabetic patients exhibit defective metabolic reprogramming, leading to suppressed functions and reduced anti-infection capabilities (Cao et al., 2024; Romero-Carramiñana et al., 2024). Metabolic abnormalities may cause T cell exhaustion and dysfunction, closely linked to chronic inflammation and autoimmune diseases. Notably, lactate accumulation can modulate the immune microenvironment, suppressing effector T cell functions while promoting regulatory T cell activity, thus influencing inflammation progression (Moraly et al., 2024).

Activated T cells play a critical role in the pathogenesis of diabetic osteomyelitis. Studies show that these cells secrete various pro-inflammatory cytokines, such as interferon-γ (IFN-γ) and interleukin-17 (IL-17), which promote local inflammatory responses in osteomyelitis, leading to bone tissue destruction. For example, IFN-γ enhances macrophage activity, exacerbating local inflammation, while IL-17 stimulates fibroblast and osteoclast proliferation and activation, accelerating bone resorption (Leney-Greene et al., 2020; Schwager et al., 2021). As pro-inflammatory cytokines increase, osteoclast activity is significantly enhanced, leading to further bone destruction and lesion expansion. Meanwhile, interactions between immune cells and bone cells exacerbate this pathological progression, forming a vicious cycle that aggravates diabetic osteomyelitis (Waugh et al., 2023; Szabó et al., 2024).

Additionally, metabolic abnormalities in diabetic patients may impair T cell function, reducing their response to infections. The hyperglycemic environment induces T cell metabolic reprogramming, affecting proliferation and effector functions, thereby weakening immune responses and increasing the risk of osteomyelitis recurrence (Yang et al., 2023; Reed et al., 2025). This chronic inflammatory state not only sustains T cell activation but also forms a self-reinforcing cycle, further aggravating the pathological state of osteomyelitis.

### 4.3 Role of immune cell metabolic reprogramming in bacterial infections of diabetic osteomyelitis

The occurrence and progression of diabetic osteomyelitis extend beyond simple bacterial infections, fundamentally representing a complex interaction between pathogens and the host immune system in a unique environment of metabolic dysregulation. The diabetic metabolic microenvironment, characterized by hyperglycemia, insulin resistance, and chronic inflammation, profoundly reprograms the metabolic homeostasis and functional states of various immune cells. This intrinsic cellular metabolic dysregulation, combined with bacterial strategies to actively manipulate host metabolism for survival, collectively leads to immune response failure and the onset of infections.

#### 4.3.1 Bacterial immune adaptation features

As previously described, diabetic osteomyelitis is accompanied by immune cell metabolic reprogramming, which limits their function in responding to infections and inflammation. On the other hand, bacteria, as a factor in osteomyelitis infections, influence host immune cell function and alter immune responses by releasing metabolic products. For instance, bacterial exogenous metabolites (e.g., the bacterial “exometabolome”) have been found to suppress host immune responses to some extent, thereby promoting bacterial survival and pathogenicity (Chugh et al., 2025). For example, short-chain fatty acids (SCFAs) and other metabolites can promote immune tolerance and anti-inflammatory responses by activating specific host immune pathways (Fang et al., 2025). Bacteria manipulate host cell metabolism to enhance their adaptability and survival in the host immune environment, such as by promoting glycolysis and inhibiting oxidative phosphorylation to regulate macrophage metabolism (Quan et al., 2023), thereby favoring bacterial growth rather than eliciting effective immune responses (Ahator et al., 2024). These findings suggest that bacterial metabolites are critical signaling molecules in regulating host immune metabolism. This immune adaptation mechanism not only enhances bacterial survival but also enables their long-term persistence in the host, leading to chronic infections.

#### 4.3.2 Macrophage metabolic reprogramming and bacterial clearance

M1 macrophages generate energy and metabolic intermediates through enhanced glycolysis to support rapid proliferation, inflammatory responses, and bacterial killing, a hallmark feature of their role as innate immune cells (Malla et al., 2025). For instance, enhanced glycolysis is closely associated with bacterial phagocytosis and subsequent killing, particularly in managing infections like *Escherichia coli*, where glycolytic metabolites are critical for macrophage antibacterial activity (Yang et al., 2021). In contrast, M2 macrophages primarily promote tissue healing and reduce excessive inflammation through anti-inflammatory cytokine secretion post-infection (Zhang Z. et al., 2024). Additionally, M2 macrophage metabolic activity is linked to bacterial clearance efficiency, particularly in chronic infection scenarios, where their metabolic reprogramming influences responses to pathogens (Lane et al., 2023). However, in diabetic patients, hyperglycemia-induced oxidative stress and inflammation disrupt the balance between M1 and M2 macrophage functions. Macrophages exhibit enhanced M1 characteristics while M2 functions are suppressed, leading to reduced anti-infection capacity (Hegde et al., 2024). This metabolic imbalance impairs macrophage antibacterial activity, increasing the risk of diabetes-related complications (Xu et al., 2025).

#### 4.3.3 T cell metabolic reprogramming and immune response

Unlike macrophages, T cells do not directly participate in bacterial killing but clear virus- or bacteria-infected cells through cellular immunity, with their activation and function heavily dependent on their metabolic state. Upon antigen stimulation, T cell metabolic pathways shift from glycolysis to oxidative phosphorylation. Specifically, effector T cells typically rely on glycolysis to meet the energy demands of rapid proliferation, while memory T cells preferentially utilize oxidative phosphorylation to maintain longevity and functionality (Levine et al., 2021).

In the pathological environment of diabetes, T cell metabolic abnormalities significantly impair their immune response capacity. Factors such as hyperglycemia, chronic inflammation, and metabolic syndrome may lead to T cell dysfunction, manifesting as reduced proliferation and cytokine production (Gao et al., 2024; Qiu et al., 2025). For instance, in diabetic mouse models, T cell metabolic reprogramming is inhibited, significantly weakening their anti-infection capacity, likely due to imbalanced metabolic competition and insufficient energy supply (Ricci, 2025). Additionally, metabolic regulation impacts the balance between regulatory T cells (Tregs) and effector T cells (Teffs). Tregs primarily rely on oxidative phosphorylation and fatty acid oxidation to maintain their suppressive functions, while Teffs enhance activity through activated glycolytic pathways (He et al., 2021; Noble et al., 2024). For example, Treg function and proliferation are influenced by their metabolic state, and excessive glycolysis may suppress Treg function, promoting autoimmune disease development (Röring et al., 2024). Diabetic osteomyelitis involves immune dysregulation, and changes in T cell metabolic reprogramming further exacerbate inflammatory responses and bone tissue damage.

#### 4.3.4 Neutrophil metabolic reprogramming and inflammatory response

Neutrophils, a critical component of the innate immune system, play key roles in combating infections and inflammatory responses. Their metabolic state directly affects chemotaxis, phagocytosis, and bactericidal capacity. Recent studies indicate that neutrophils exhibit diverse metabolic adaptability beyond traditional glycolysis. In inflammatory microenvironments, where oxygen and nutrients are limited, neutrophils must adjust their metabolic pathways to maintain function. Studies have found that neutrophils at inflammatory sites reprogram their metabolic pathways to generate energy and support antibacterial functions (Morrison et al., 2023). For instance, mitochondrial metabolism plays a significant role in supporting neutrophil migration, neutrophil extracellular trap (NET) formation, and bacterial killing (Maldarelli and Noto, 2024). Upon activation, neutrophils rapidly reprogram their metabolic pathways to enhance pathogen responses. This metabolic reprogramming not only affects energy production but also alters the nature of their inflammatory responses, enabling task execution in diverse microenvironments. In chronic disease states like diabetes, neutrophil glycolysis and oxidative phosphorylation are impaired, suppressing their metabolic functions, reducing antibacterial capacity, and inducing infections (Holder et al., 2025). Concurrently, neutrophil metabolic abnormalities not only weaken their function but also exacerbate chronic inflammation, forming a vicious cycle.

#### 4.3.4 Potential significance of the microbiome–metabolism–immunity axis in the treatment of DO

In recent years, studies have gradually revealed the relationship between the gut microbiota and bone metabolism, referred to as the “gut–bone axis.” Evidence indicates that microorganisms and their metabolites not only affect the local intestinal environment but also regulate immune responses and metabolic balance in bone tissue through immune, endocrine, and metabolic pathways, thereby influencing bone health and disease progression (Grüner et al., 2023; Chen Y. et al., 2025). For example, postmenopausal osteoporosis (PMOP), which is closely associated with estrogen deficiency, is also characterized by dysbiosis of the gut microbiota and imbalance in Th17/Treg ratios. Short-chain fatty acids (SCFAs) regulate T-cell differentiation through specific receptors, suggesting that modulation of the microbiota and its metabolites may provide novel strategies for PMOP therapy (Chen Y. et al., 2025). Moreover, gut microbial diversity and functional status directly affect bone mineral density and remodeling, and in patients with inflammatory bowel disease (IBD), dysbiosis is strongly associated with bone-related complications such as osteoporosis and arthritis (Grüner et al., 2023).

Microbial metabolites such as SCFAs, bile acids, and tryptophan derivatives can reshape immune cell metabolism and regulate their functions (Michaudel and Sokol, 2020; Wang et al., 2023). Specifically, SCFAs promote Treg differentiation while suppressing pro-inflammatory cells; bile acid metabolites (e.g., GLCA) enhance Treg proliferation via nuclear receptors and induce osteogenic differentiation of bone marrow mesenchymal stem cells (Cai et al., 2024); tryptophan derivatives act through the aryl hydrocarbon receptor (AHR) to improve gut barrier function and modulate immune responses (Fu et al., 2023). In addition, both gut and oral microbiota can regulate the local bone immune environment via the “gut–bone axis.” Their metabolites may translocate to bone tissue, leading to aberrant immune cell activation, bone resorption, and inflammation (Jia et al., 2021; Han et al., 2023).

More recently, increasing attention has been paid to the presence and characteristics of local bone microbiota. Although bone was traditionally considered a sterile environment, accumulating evidence suggests otherwise. The bone microbiota displays considerable diversity, including major bacterial phyla such as Proteobacteria, Actinobacteria, Firmicutes, and Bacteroidetes (Emmons et al., 2020). These microbes may contribute to the maintenance of the local bone environment and, through their metabolites, regulate the bone immune microenvironment. Metabolic activities within bone generate bioactive molecules—such as SCFAs, bile acid derivatives, and tryptophan metabolites—that influence local immune cell function and bone metabolic processes (Hansdah and Lui, 2024; Qiao et al., 2025).

Bone microbial metabolites modulate the activity of immune cells such as T cells, macrophages, and bone marrow mesenchymal stem cells (BM-MSCs), thereby shaping the bone immune microenvironment. For example, butyrate promotes Treg expansion and suppresses inflammation, protecting bone tissue from excessive inflammatory damage (Kermgard et al., 2021; He et al., 2025). Certain microbial metabolites, such as deoxycholic acid, regulate hematopoietic progenitors in the bone marrow, enhancing monocyte numbers and function to support immune homeostasis (Burgess et al., 2020). Conversely, dysbiosis of the local bone microbiota may create a pro-inflammatory milieu, promoting bone resorption and contributing to bone metabolic disorders and inflammatory bone diseases (Han et al., 2023; Dmytrenko et al., 2024).

The interactions between the bone microbiota, metabolism, and inflammation underscore the complexity of the microbiota–immune–bone metabolism axis. Microorganisms and their metabolites within bone tissue regulate local immune responses, thereby influencing the balance between bone formation and resorption. For instance, gut microbiota-derived metabolites can indirectly affect bone mineral density and strength (Castaneda et al., 2020; Luna et al., 2021). Meanwhile, bone-resident microbiota may activate immune cells to release pro- or anti-inflammatory factors, modulating bone matrix remodeling (Pianko and Golob, 2022). In pathological states such as osteoporosis, bone metastases, and bone marrow disorders, changes in the composition and function of the bone microbiota are closely linked to immune dysregulation, suggesting a potential regulatory role in the pathogenesis of bone diseases (Lian et al., 2022; Sevcikova et al., 2024).

In summary, gut and bone microbiota, together with their metabolites, regulate immune cell metabolic reprogramming through complex signaling and metabolic networks. They are critical for maintaining immune homeostasis, modulating inflammation, and controlling bone metabolism. Elucidating these mechanisms not only highlights the role of the “gut–bone axis” in health and disease but also provides a theoretical basis for developing therapeutic strategies targeting microbiota and their metabolites (Grüner et al., 2023; Chen Y. et al., 2025).

## 5 Immunomodulatory perspective on the treatment and outlook for diabetic osteomyelitis

### 5.1 Current treatment strategies and their limitations

Given that the core pathogenesis of diabetic foot osteomyelitis (DFO) involves immunosuppression and bacterial infections induced by hyperglycemia, clinical management of DFO primarily focuses on glycemic control, antibiotic therapy, and surgical debridement, with antibiotic therapy being the most critical. DFO is typically caused by mixed infections involving multiple pathogens, commonly including *Staphylococcus aureus* and other Gram-positive and Gram-negative bacteria, making the antimicrobial spectrum of antibiotics a cornerstone of treatment (Aicale et al., 2020). Beyond traditional antibiotic combinations, the use of anti-biofilm antibiotics, such as rifampin, significantly improves healing outcomes and reduces the risk of infection recurrence (Senneville et al., 2023). Antibiotic-impregnated bone cement can also be used during tissue reconstruction, effectively shortening healing time, hospital stays, and infection recurrence rates (Mendame Ehya et al., 2021). Some clinical cases demonstrate that combining these novel therapies with traditional antibiotics can effectively reduce hospitalization time and amputation rates (Hassanin et al., 2025). The widespread use of novel drugs like dalbavancin effectively controls multidrug-resistant bacterial infections and reduces hospitalization duration (Loupa et al., 2020). Additionally, studies have found that rifampin, as an adjuvant therapy, significantly improves healing rates in diabetic foot osteomyelitis and offers better efficacy than traditional treatments (Bessesen et al., 2020). However, drug interactions and patient comorbidities limit its clinical application, necessitating further research to explore safer alternatives, such as rifabutin (Mallarino-Haeger et al., 2024). Notably, the lack of high-quality clinical trial data for medical and surgical treatments of diabetic foot osteomyelitis poses challenges in selecting optimal treatment strategies (Tardáguila-García et al., 2021).

The pathogenesis of DO is rooted in hyperglycemia, and the chronic low-grade inflammatory (CLGI) state in patients continuously increases infection risk and delays wound healing. Therefore, glycemic control is essential during DO treatment to prevent complications (Gramberg et al., 2024). Poor glycemic control exacerbates DO clinical symptoms (Nilesh et al., 2022). Interventions targeting the metabolic state of diabetic patients can reduce inflammation in osteomyelitis, thereby promoting healing (Pakkiyaretnam and Chong, 2023). Compared to general infections, DO often accelerates gangrene development, necessitating surgical debridement in severe cases to remove infected bone tissue, prevent infection spread, and promote healing (Jing et al., 2025). Early surgical intervention significantly improves DO prognosis (Venkatesan and Rangasamy, 2023). Among patients undergoing surgical debridement, approximately 93.6% achieve complete healing, with only a minority requiring amputation (Moosa et al., 2023).

Overall, traditional antibiotic therapy and surgical debridement remain the primary methods for DO management, but immunomodulatory and metabolic regulation approaches offer new perspectives and possibilities for improving patient outcomes. These approaches demonstrate potential to enhance efficacy when combined with traditional treatments (Meyer-Schwickerath et al., 2023), representing a critical entry point for future DO therapies.

### 5.2 Immunomodulatory therapies for diabetic osteomyelitis

As previously described, although DFO manifests as bacterial infections, its core pathology stems from immune system dysfunction caused by the CLGI state. From an immunomodulatory perspective, suppressing chronic inflammatory damage and enhancing immune responses can potentially improve DFO prognosis. Existing studies show that glucocorticoids reduce inflammation by suppressing immune cell activity, thereby lowering infection susceptibility (Lipsky et al., 2012). Biologics achieve immunomodulation by neutralizing specific pro-inflammatory cytokines (Mens et al., 2023). A retrospective study reported that diabetic foot osteomyelitis patients treated with bioactive glass (S53P4) achieved significantly higher healing rates than those receiving conventional treatment (90% vs. 61.9%) (De Giglio et al., 2021). Patients treated with small-molecule immunomodulatory agents exhibited better infection control and faster healing compared to controls (Zhang et al., 2021), highlighting their value in managing refractory infections. Notably, current immunomodulatory agents for diabetes primarily target type 1 diabetes (T1D) [summarized in **Table 2** (Ajmal et al., 2024)]. Few immunomodulatory agents are available for type 2 diabetes mellitus (T2DM), significantly limiting their application in DFO treatment. This is because T1D is an autoimmune disease characterized by T-cell-mediated specific attacks on pancreatic β-cells, allowing precise interventions like anti-CD3 monoclonal antibodies or antigen vaccines to delay or prevent onset (Mauvais and van Endert, 2025). In contrast, T2DM is driven by metabolic syndrome, with insulin resistance and β-cell failure, where inflammation is a chronic, low-grade, multi-cytokine “background noise” without a single immune target or clear biomarkers like autoantibodies. As described earlier, the CLGI state in T2DM creates a complex interplay of excessive inflammation and immunosuppression, which cannot be simply managed with immune agonists or suppressors. Moreover, immunomodulation carries risks of increased infections, tumors, and cardiovascular events, particularly in chronic conditions like DFO requiring long-term intervention, further limiting the clinical application and development of immunomodulatory therapies. Nevertheless, their potential therapeutic benefits have prompted numerous research efforts.

**Table 2:** Investigational new drugs with immunomodulatory properties.

Drug name(s)	Target	Side effects	NCT number(s)	Published results
Teplizumab (Tzield)	Anti-CD3 monoclonal antibody	• Headache • Gastrointestinal issues • Lymphopenia • Mild cytokine release syndrome (CRS)	NCT00385697 NCT04598893 NCT03875729 NCT05757713	Hagopian et al., 2013
Otelixizumab (TRX4)	Anti-CD3 monoclonal antibody	• Headache • Gastrointestinal issues • Arthralgia • Myalgia	NCT00678886 NCT01123083	Aronson et al., 2014 Keymeulen et al., 2005
Daclizumab (Zinbryta, Zenapax)	Anti-CD25 monoclonal antibody	•Gastrointestinal infections •Neutropenia and leukopenia • Elevated liver enzymes • Hypoglycemia	NCT00064714 NCT00468117	Rother et al., 2009
Ladarixin	Inhibitor of IL-8 receptors (CXCR1 and CXCR2)	Gastrointestinal infections Dyspepsia • Headache	NCT04628481	None
Antithymocyte globulin (ATG) (Thymoglobulin, Atgam)	T lymphocyte depletion	• Fever • Headache • Nausea • Lymphopenia • Serum sickness	NCT01106157 NCT02215200 NCT00434811 NCT00468117	Haller et al., 2015 Foster et al., 2018

For instance, N-acetylcysteine (NAC), an immunometabolic regulator, significantly reduces inflammation in DFO patients and accelerates antibiotic treatment responses (Hooshmand Gharabagh et al., 2025). Other metabolism-related biomarkers, such as serum lipids and amino acids, can serve as monitoring indicators to guide clinical decision-making and improve DFO management (Boucher et al., 2024; Roh et al., 2024). Clinical trials have also explored personalized immunomodulatory therapies for DFO. For example, a diabetic cohort study showed that patients receiving small-molecule immunomodulatory agents had superior infection control and healing rates compared to controls (Osório et al., 2023). However, the complex immune characteristics of T2DM significantly increase the difficulty of DFO immunotherapy, making “risk-benefit” assessments critical for clinical decision-making.

Notably, leveraging the immune characteristics of DFO, potential treatment approaches are being explored and optimized. Nanodrug delivery systems enhance targeting and biocompatibility, with nanoparticle carriers enabling localized delivery of immunomodulatory agents to infection sites while minimizing systemic exposure. Gene-editing tools like CRISPR show promise in correcting diabetes-related immune defects, with preclinical studies indicating improved antibacterial responses and reduced osteomyelitis incidence (Tubin et al., 2023). Cell therapies also hold translational potential. Transplantation of umbilical cord blood or bone marrow-derived mesenchymal stem cells (MSCs) reshapes the immune microenvironment and enhances anti-infection capacity by secreting trophic factors, thereby improving osteogenesis in diabetic patients (Yan, 2024; Freitas et al., 2025). However, these approaches require extensive preclinical validation before clinical use, and systemic immunotherapies for DFO remain largely conceptual. In contrast, interventions targeting metabolic reprogramming are relatively simple and controllable. Modulating the metabolic characteristics of the DFO environment to influence downstream immune cell functions represents a promising approach to address the complex immune background of DFO.

### 5.3 Metabolic interventions for diabetic osteomyelitis

The core pathogenesis of DFO is the CLGI state under a hyperglycemic background, making glycemic stability a primary goal in treating diabetic patients, particularly those with osteomyelitis. Studies indicate that good glycemic control reduces bone infection risk and promotes healing in diabetic patients (Khandelwal et al., 2022; Jaroenarpornwatana et al., 2023). Additionally, maintaining glycemic stability reduces postoperative complications, enhances recovery, and lowers infection rates. Beyond direct pharmacological interventions, auxiliary metabolic regulation therapies, such as diet, exercise, and hyperbaric oxygen therapy, are critical for alleviating DFO clinical symptoms.

Balanced diets improve the metabolic state of diabetic patients, reducing bone infection risk. High-fiber, low-sugar, low-fat dietary patterns are foundational for maintaining healthy blood glucose levels. For instance, the Mediterranean diet, characterized by abundant fruits, vegetables, whole grains, and healthy fats, has been shown to improve metabolic markers in diabetic patients, reducing cardiovascular disease and bone infection risks (Szymczak et al., 2023). Nutritional interventions, such as vitamin D and calcium supplementation, effectively promote bone healing—vitamin D not only aids calcium absorption but also enhances osteoblast function. Thus, personalized dietary plans tailored to the specific needs of diabetic patients are critical strategies for preventing bone infections (Iles et al., 2021). Additionally, moderate exercise effectively improves metabolic markers in diabetic patients (Silva et al., 2024), enhances blood circulation, and boosts immune function (Seidu et al., 2021). Exercise also stimulates bone cell proliferation and differentiation, increasing bone mechanical strength (Chang et al., 2022), which is beneficial for accelerating DFO healing. Furthermore, exercise reduces chronic inflammation levels, mitigating diabetes-related osteoporosis and bone infection risks. For example, studies show that diabetic patients engaging in regular weight-bearing exercise exhibit significantly higher bone density and strength than non-exercisers, highlighting exercise’s critical role in bone health (Kawanishi et al., 2022; D’Haese et al., 2024). Blood oxygen metabolism is another key approach for improving DO. Diabetic patients often experience microvascular complications, leading to poor blood circulation and impaired bone tissue blood supply and nutrient delivery. Local treatments using bioactive materials like bioactive glass enhance local blood circulation, improve oxygenation, and accelerate bone healing (De Giglio et al., 2021; Zhang et al., 2021). The classic oxygen metabolism regulation method is hyperbaric oxygen therapy (HBOT), where high-concentration oxygen improves tissue oxygenation and promotes angiogenesis. In diabetic patients, HBOT effectively increases local oxygen partial pressure, improves microcirculation, and reduces infection occurrence and progression. Despite its lower applicability, higher costs, and significant individual variability, HBOT remains an important method for regulating blood oxygen metabolism (Huang et al., 2021).

## 6 Conclusion

As our understanding of the mechanisms behind immunometabolic reprogramming deepens, researchers have increasingly recognized that immune cells in diabetic patients often exhibit metabolic dysfunction when confronted with infections. This dysfunction not only impairs the immune system’s ability to combat pathogens but also hinders the regeneration of bone tissue. This understanding is crucial in diabetic osteomyelitis, as the metabolic state of immune cells directly influences the inflammatory response, infection resolution, and bone healing. Current research has highlighted the involvement of multiple signaling pathways and metabolic routes in this process. Key factors such as inflammatory cytokines, oxidative stress, and nutritional status influence immunometabolic reprogramming, leading to chronic inflammation and impaired immune cell function. In clinical practice, these factors must be considered to develop more effective treatment strategies. While traditional therapies, such as antibiotics and surgical debridement, continue to play a pivotal role, they often fall short in addressing the underlying immune and metabolic dysfunctions that exacerbate diabetic osteomyelitis. Emerging treatments that target specific immunometabolic pathways offer significant promise. For example, immunomodulatory therapies that regulate immune responses and metabolic interventions aimed at restoring normal immune cell function may complement traditional therapies. These strategies represent a promising direction for future clinical approaches, which could improve patient outcomes by addressing both the infection and the underlying immune-metabolic disturbances. However, the complexity of type 2 diabetes mellitus (T2DM) and its associated chronic low-grade inflammation pose challenges in developing and implementing these novel therapies. The immunosuppressive environment in diabetic patients complicates the identification of specific therapeutic targets and increases the difficulty of achieving long-term efficacy. Thus, future clinical research should focus on personalized treatment strategies that integrate both metabolic and immunomodulatory approaches. Multidisciplinary collaboration and large-scale, randomized clinical trials will be critical in validating these new treatments and translating laboratory findings into effective clinical practices. By bridging the gap between basic research and clinical applications, we can enhance our ability to manage and treat diabetic osteomyelitis, improving both the quality of life and clinical outcomes for patients.
